# Chemical Theory or Chemical Philosophy

**Published:** 1881-03

**Authors:** J. U. Lloyd


### CHEMICAL THEORY, OR CHEMICAL PHILOSOPHY.

BY J. U. LLOYD.

The origin of the word *chemistry* is involved in obscurity. Some believe it to have reference to the soil of Egypt, and to denote something black. Thus we hear the early science spoken of as the *black art*, other and equally good authorities believe that the word *chemistry* was derived in early days from a word which signified *heat*, and this also seems probable, inasmuch as the majority of chemical operations require the heat.

Chemistry at the present day may be defined as that branch of natural science which treats of the properties of simple and of compound matter; of the relations of the various elements; of the compounds which may be formed of primary matters, and of the laws which influence combination (synthesis), and decomposition (analysis).

MATTER.--What has weight or occupies space is matter. Thus we may include in the same class hydrogen gas, the lightest, and osmium, the heaviest of the known elements. All material of this world is a form of matter, and its characteristic property is in its indestructibility. Man admits that he can not destroy the smallest particle of matter, neither can he by any known power create. He has never noted the production of matter by an act of Nature, and Nature has never been known to annihilate. Man can bring no scientific evidence to disprove the assertion that all matter existed from the beginning, and will continue to exist forever.

FORMS OF MATTER.—While it is admitted that matter is imperishable, we all know that many *forms* of matter exist. Thus we have iron, copper, gold, silver, lead, etc. These are primary forms of matter. They are classed under the general name, *matter*, but each differs from the other. The several substances have distinct properties, and this immutability man has never been able to overcome. *

PRIMARY FORMS OF MATTER, OR THE ELEMENTS. — All bodies that have never been decomposed into simpler forms of matter are called *primary bodies* or *elements*. There are at present sixty-four known elements.

ALLOTROPY.—When this word is applied to an element we understand that the element is capable of existing in two or more distinct conditions. There is no change in composition, but the

* It must be remembered that we speak with the light which is now before us. At one time some of our common salts were called elements. The knowledge of a few years may prove that our elements are compounds. Indeed, the indications are that chemists may yet possess the power to decompose many of our so-called elements. However, if this should be so, and if all matter should be proven a modification of force, the chemist and theorist will be confronted with the problem as to the origin of force.

chemical and physical properties of the various modifications differ to a more or less extent. As an illustration, we ask the reader to refer to phosphorus. This element may be made to assume three distinct forms, each of which differs from the others in a remarkable manner. The diamond, plumbago, and ordinary charcoal are all carbon. Sulphur exists in several allotropic forms, yet each form is sulphur. When an element assumes an allotropic form, we consider it simply a modification of the original body, regardless of the change in both chemical and physical properties. This change in the condition of an element is generally accepted as the result of a difference in the arrangement of its atoms.

Constitution of Primary Matter.—We have referred to the elements as though they were homogeneous bodies. We must now go further and assume that any perceptible amount of an element is capable of subdivision, until a given point is reached. This we consider the smallest particle of an element—*the atom*—and we accept that each form of primary matter is composed of atoms.

Atoms.—An atom is understood to be the smallest particle of an element capable of taking part in a chemical reaction. We have no means of judging of the size of atoms. We simply assume that such bodies exist, and consider that each perceptible part of an element is made up of an innumerable number of atoms. Copper is supposed to be made up of atoms which have properties that distinguish the mass of copper from any other form of primary matter. This is true of gold, silver, oxygen, and the entire list of elementary bodies. We thus accept the old, old theory, that all matter is made up of atoms. We may to-day accept for the definition of the atoms, and perhaps not improve upon it, that made by Demokritos, 460 B. C., viz: "The atoms are invisible by reason of their smallness; indivisible by reason of their solidity; impenetrable and unalterable."

Atomatic Weight.—In all works upon chemistry at the present day, we find after each element a number which expresses the atomic weight of that element, and which might be mistaken by the student for the *actual* weight of the atom of the element. The atomic weight of an element is accepted by chemists as the weight of an atom of that element, compared with an atom of hydrogen, but we have no means of determining the absolute weight of an atom of hydrogen, and we therefore do not know the actual weight of an atom of any element. As examples of atomic weight we give:

| | |
|---|---|
| Antimony | 122 |
| Uranium | 240 |
| Copper | 63 |

The above indicates that an atom of antimony is 122 times as heavy as an atom of hydrogen. An atom of uranium is 240, and an atom of copper is 63 times as heavy as an atom of hydrogen. Hydrogen is the lightest known body, the atom of hydrogen is accepted as unity (1). We may then define *atomic weight*, as the weight of the atom of any element, compared with an atom of hydrogen. .

COMBINATIONS OF ATOMS (MOLECULES).—The atoms of elementary bodies are not believed to be in a free state. We consider that a bond (chemism) unites a certain number of atoms, and this combination of the atoms of an element is called the *elementary molecule.* This molecule, then, is a combination of atoms which are alike.* The elementary molecule is supposed to be the smallest particle of an element that can exist in a free state.

BREAKING UP OF THE MOLECULE.—The atoms of an element are the indivisible constituents of its molecules. We have said that atoms do not (according to our present view) exist in a free state, excepting the few which are said to play the part of molecules. (See note below.) The breaking up, then, of the molecule of the element must destroy the properties of the element. Example: The molecu'e of chlorine consists of two atoms of a kind, and the molecule of hydrogen also consists of

* According to rather recent investigations the molecule of mercury and of nitrogen, each consists of one atom. Accepting this view we may say that the elementary molecule is the smallest particle of an element that can exist in a free state and perform the part of the element. This particle consists of one or more atoms, united in the latter case by chemism.

two atoms, thus: HH represents the hydrogen molecule; ClCl represents the chlorine molecule.

The above combination of atoms are elementary molecules, because they are formed in the one case by atoms of hydrogen, and in the other case by atoms of chlorine. Let us bring any even number of these molecules together in the sunlight and the positions of the atoms will be altered. An atom of chlorine unites with an atom of hydrogen, the result being two molecules in which the atoms are *unlike*, HCl and HCl. Each of these molecules is a *compound molecule*, and the properties of the original bodies will have disappeared.*

COMPOUND MOLECULES may be then defined as the smallest particle of a *compound*, and all compounds are composed of atoms that are unlike. We have for example:

| | |
|---|---|
| Chloride of Sodium.................... | NaCl. |
| Chloride of Potassium..................... | KCl. |
| Chloride of Hydrogen...................... | HCl. |

and each molecule contains an atom of two different elements. We thus pass from the study of the primary form of matter to the study of—

COMPOUND MATTER.—The smallest particle of compound matter is a *molecule*. There can be no *atom* of any compound, for all compounds contain two or more kinds of primary matter, and therefore, the smallest conceivable portion of any compound must contain at least two atoms. As an example, we refer to bromide of potassium. Into any given amount of this compound the element bromine and the element potassium must enter. If we imagine that we have one hundred molecules of bromide of potassium, each molecule must contain one atom of bromine and one atom of potassium. We can separate the piece of potassium bromide into two parts, but each part will consist of molecules. We may theoretically subdivide the fragments into the one hundred molecules, and each molecule will have the properties of the

*Some speak of compounds containing elements. Elements enter into compounds and may be again obtained from the compound. The element, however, loses its individuality when it passes into combination with another.

original article, and further division would result in the production of atoms of bromine and atoms of potassium.

PRIMARY MATTER AND COMPOUND MATTER DEFINED.—

| *Primary Matter.* | *Compound Matter.* |
| --- | --- |
| Atoms of one kind of primary matter unite to form elementary molecules. | Atoms of different kinds of primary matter unite to form compound matter. |
| Primary matter consists of atoms that are unlike, united by chemism. | Compound matter consists of atoms of two or more kinds of primary matter, united by chemism. |
| The smallest portion of primary matter is the atom. | The smallest portion of compound matter is the molecule. |

CHEMISM.—There is an intimate connection between chemism, heat, electricity, and other forces, which is thoroughly discussed in works upon physics. This is a name which is given to the force which we believe produces the changes in the position of atoms. Chemical attraction, chemical affinity, and chemical force, are terms employed by chemists, denoting the same object, and all refer to this force. Chemism is known by its effects. The constitutional transformation which is constantly taking place in matter, we credit to the action of this force. All forms of matter are subservient to it. The changes which it produces are infinite in number. Our very bodies grow and waste in obedience to this power, and yet the real force we denominate *chemism* is unknown.

EFFECTS OF CHEMISM---Chemical force destroys the distinctive properties of bodies, producing new substances. When elements unite, the resulting compound is different from either of the elements. For example, oxygen and hydrogen gases unite to form the liquid, water. If we heat sulphur and iron together, an entirely different substance results. In neither of the foregoing cases could we judge of the properties of the compound by the character of the elements which enter into it.

The antithesis of combination is decomposition; both depend upon the action of chemical force. Thus it is that from substances which are decomposed, bodies entirely different from the original may arise. Place, for example, a piece of sugar in a spoon, and hold it in the flame of an alcohol lamp. The sugar is decomposed, and the result will be a number of substances, none of which resemble sugar in the least. When a candle burns the tallow changes by combination with oxygen into (mostly) water and carbon dioxide, both of which are colorless gases, and differ from tallow and the oxygen of air in every way. Let it be remembered then that the result of chemical action, is altering of the properties of the substances concerned, with production of new bodies which have characters entirely distinct from those of the original.—*Chemistry of Medicine.*

